# Novel detection method for chemiluminescence derived from the Kinase-Glo luminescent kinase assay platform: Advantages over traditional microplate luminometers

**DOI:** 10.1016/j.mex.2014.07.003

**Published:** 2014-08-08

**Authors:** Ryan A.V. Bell, Kenneth B. Storey

**Affiliations:** Institute of Biochemistry and Department of Chemistry, Carleton University, 1125 Colonel By Drive, Ottawa, Ontario, Canada K1S 5B6

**Keywords:** Protein kinase assay, Kinase-Glo, Microplate luminescence detection

## Abstract

The efficacy of cellular signal transduction is of paramount importance for the proper functioning of a cell and an organism as a whole. Protein kinases are responsible for much of this transmission and thus have been the focal point of extensive research. While there are numerous commercially available protein kinase assays, the Kinase-Glo luminescent kinase assay (Promega) provides an easy-to-use and high throughput platform for determining protein kinase activity. This assay is said to require the use of a microplate spectrophotometer capable of detecting a luminescent signal. This study shows that:

• The ChemiGenius Bioimaging system (Syngene), typically used for visualizing chemiluminescence from Western blots, provides an alternative detection system for Kinase-Glo luminescence.
• The novel detection system confers an advantage over traditional luminometers, in that it allows visualization of the luminescent wells, which allows for the real-time analysis and correction of experimental errors (i.e. bubble formation).
• Determining kinase kinetics using this detection system produced comparable results to previous studies on the same enzyme (i.e. glycogen synthase kinase 3).

## Graphical abstract

**Figure:**
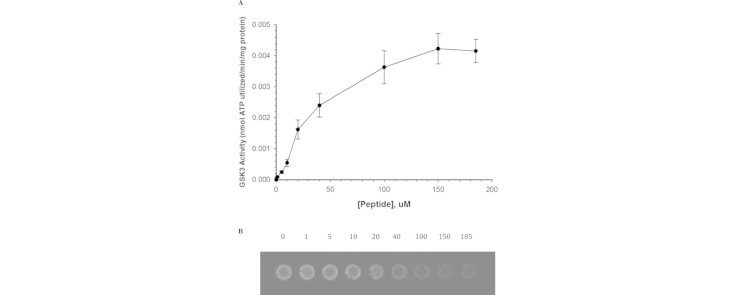

## Materials and methods

### Kinase-Glo standard curve

ATP standard curves were created using both the Cary Eclipse Fluorescence Spectrophotometer (Agilent Technologies, ON, Canada) and the ChemiGenius Bioimaging System (Syngene, MD, USA). Using either machine, Mg-ATP (made in Tris–HCl, pH 7.5) concentrations were varied between 0 and 100 μM (as suggested by the Kinase-Glo Plus platform protocol), and made up to a final volume of 50 μL in a 96-well Costar opaque black microplate or to a final volume of 20 μL in a 384-well Costar opaque black microplate. Equal volumes of Kinase-Glo Plus reagent was added to the wells containing ATP and these solutions were allowed to sit for 10 min to allow for the luminescent signal to stabilize. Detection using the Cary Eclipse spectrophotometer involved selecting single read mode for a luminescent signal at 550 nm (experimentally determined luminescent *λ*_max_) and detecting that signal for 1 s. In contrast, utilizing the ChemiGenius Bioimaging system, no specific filter was chosen and luminescence was measured for 1 s. Accompanying GeneTools software was used to measure the luminescence of each well.

### Method validation using glycogen synthase kinase assays

Kinetic assays using a skeletal muscle-derived protein kinase, GSK3, were conducted using the ChemiGenius Bioimaging System. To do so, GSK3 was partially purified from the skeletal muscle of 13-lined ground squirrels using ion exchange and affinity chromatography (specifics of purification procedure given in Appendix A).

Those fractions that contained GSK3 were subsequently pooled and spun in centrifugal filters (10 kDa cut-off; Amicon) at 8000 RPM for 20 min at 5 °C. The resulting concentrate was then used in GSK3 assays, which contained 50 μM ATP, 5 mM MgCl_2_, GSK3 peptide (YRRAAVPPSPSLSRHSSPHQ(pS) EDEEE; SignalChem) ranging from 0 to 185 μM, and 25 mM Tris–HCl, pH 7.5. The peptide is phosphorylated at the indicated serine residue as GSK3 will preferentially phosphorylate substrates that are previously phosphorylated by other kinases. The underlined serine residues in the above peptide can both be phosphorylated by GSK3 [1]. Selective addition of 1 mM LiCl was used to inhibit GSK3 activity and act as a negative control. Assays were attempted in both 96-well and 384-well Costar black microplates, and kinase reaction was allowed to proceed for 1 h at room temperature prior to the addition of the Kinase-Glo reagent. Again, once the Kinase-Glo reagent was added, the reaction wells were allowed to sit for 10 min so that the luminescent signal stabilizes. Luminescence was detected as mentioned above for the ATP standard curve.

### GSK3 activity and *K*_m_ calculation

Luminescent units determined using the GeneTools software were converted to GSK3 activities by first determining the concentration of ATP that remained after the kinase reaction. This is subsequently converted to the concentration of ATP utilized in the kinase reaction. This value was then divided by the assay time and the amount of protein present in each assay. Protein content of enzyme preparations was determined with the Bradford assay using the Bio-Rad prepared reagent and bovine serum albumin as the standard. These activities were then used to determine the *K*_m_ peptide with the help of the Kinetics v.3.5.1 computer program [2].

## Additional information

The Kinase-Glo luminescent kinase assay (Promega) is a high-throughput method for detecting protein kinase activity by quantifying the amount of ATP remaining following a kinase reaction. The luminescent signal is produced via the reaction shown below:

$$\begin{array}{l} \text{Protein}\;\text{kinase}\;\text{substrate}+\text{ATP}\to \text{Phosphorylated}\;\text{product}+\text{ADP}\\ \text{Luciferin}+\text{ATP}+½{\text{O}}_{2}\to \text{Oxyluciferin}+\text{AMP}+{\text{CO}}_{2}+{\text{PP}}_{i}+\text{Light} \end{array}$$

Typically these assays are conducted in opaque microplates with luminescence being detected using a microplate spectrophotometer capable of detecting a luminescent signal (for recent examples see [3,4]). If not already present within your laboratory, purchase of such a spectrophotometer would be significantly costly. For this reason, alternative detection systems may be extremely useful in broadening the use of this assay platform, especially if this equipment is common to many labs. The ChemiGenius Bioimaging system (Syngene) is typically used in detecting fluorescent or chemiluminescent signals from Western blots or electrophoretically separated PCR products (for recent examples see [5,6]). This study adapts the ChemiGenius Bioimaging system for the Kinase-Glo platform, validates its use for determining protein kinase activity, and indicates the advantages of using this Bioimaging system over other luminometers.

**Fig. 1:**
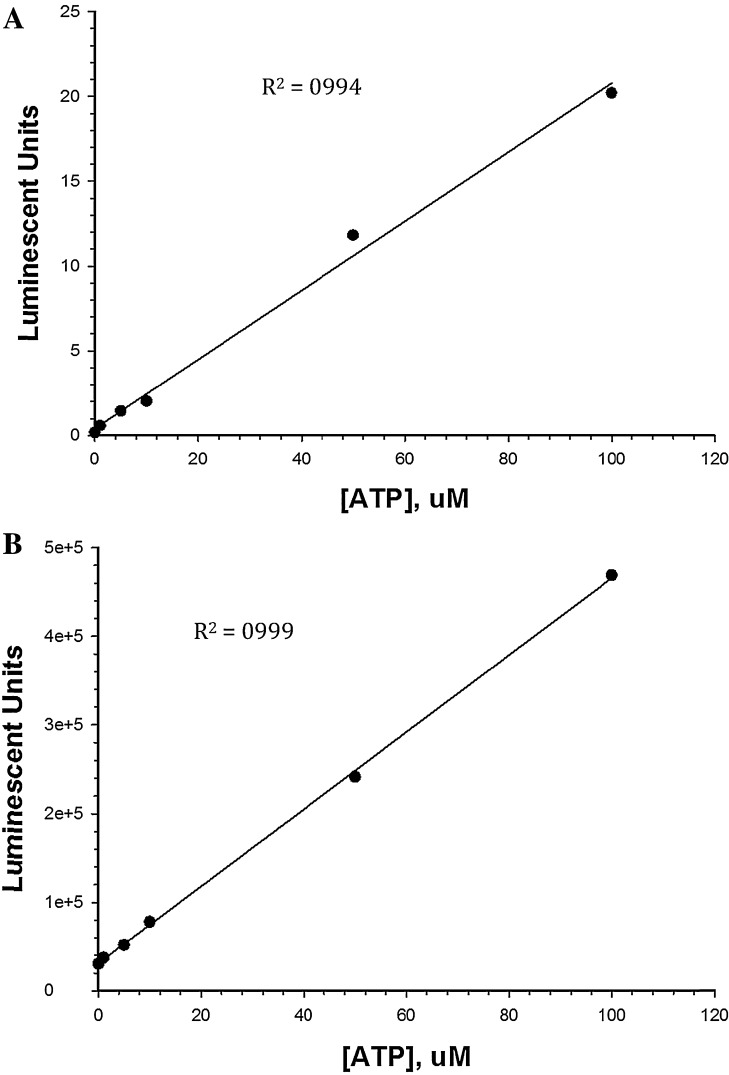
ATP standard curves for Kinase-Glo Plus luminescent kinase assay platform. (A) The ATP standard curve when utilizing the Cary Eclipse fluorescent spectrophotometer, and (B) the ATP standard curve when utilizing the ChemiGenius Bioimaging system. All data were collected using 96-well black microplates. Data are mean ± SEM, *n* = 3.

Utilization of the ChemiGenius Bioimaging system for the purpose of detecting the luminescent signal produced by the Kinase-Glo luminescent kinase assay requires that the assay will function as it would in a traditional luminometer. The first step in assessing assay suitability is to determine the linear dynamic range for the assay using the ChemiGenius Bioimaging system as compared to that observed with a luminometer. The Kinase-Glo Plus assay platform claims to have a linear dynamic range from 0 to 100 μM ATP and this is reflected in standard curves created using the Cary Eclipse fluorescence spectrophotometer and the ChemiGenius Bioimaging system (Fig. 1). Both standard curves elicited *R*^2^ values above 0.99. Given the linearity of the Kinase-Glo Plus assay platform, kinetic analyses were undertaken for GSK3 to determine the suitability of the ChemiGenius Bioimaging system for kinase assays.

**Fig. 2:**
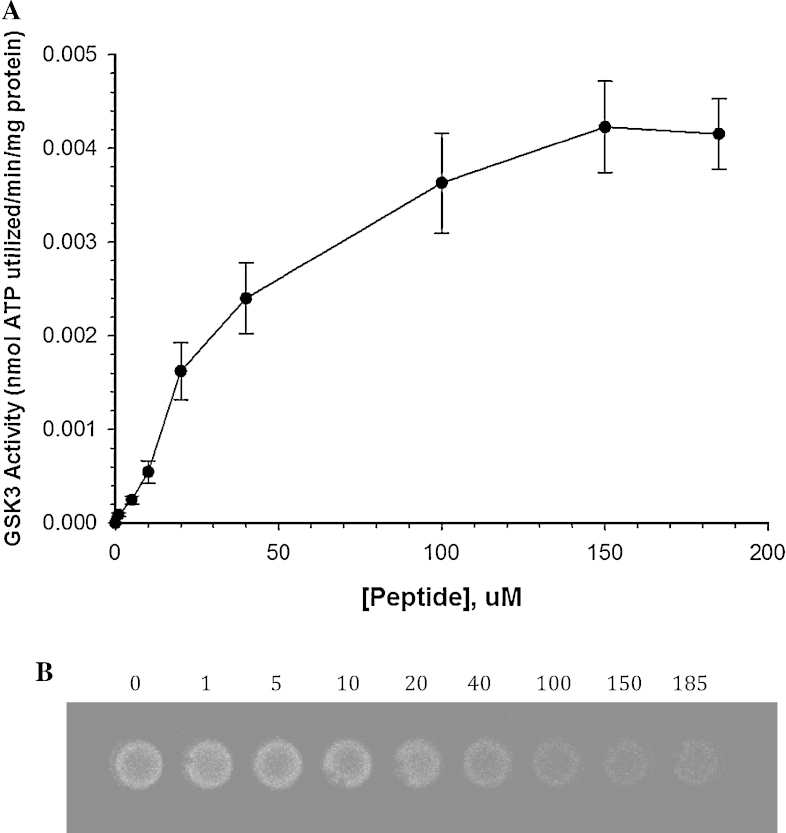
*K*_m_ peptide for GSK3 derived from the skeletal muscle of 13-lined ground squirrels. (A) The kinetic curve for the determination of *K*_m_ GSK3 peptide and (B) A representative sequence of luminescent wells that were used to generate kinetic curves shown in (A). All data were collected using 96-well black microplates. The data representative in graph are mean ± SEM, *n* = 3 on independent determinations on separate enzyme samples.

GSK3 was partially isolated from the skeletal muscle of 13-lined ground squirrels using a combination of ion exchange and affinity chromatography. GSK3 *K*_m_ peptide was subsequently determined to be 39 ± 4 μM when using the ChemiGenius Bioimaging system for luminescence detection (Fig. 2). The *K*_m_ obtained in this study is similar to those determined in studies that utilized other detection techniques. For instance, a study of wood frog GSK3 found the *K*_m_ peptide to be 23 ± 4 μM when utilizing radioactive ATP and subsequent detection of radiolabelled peptides on a phosphor screen [7]. In addition to the seemingly accurate *K*_m_ curve generated, the ChemiGenius Bioimaging system provided a unique ability to visualize the end point luminescence after the kinase reaction (Fig. 2B). This advantage over traditional luminometers allows for well-by-well assessment of experimental error, and for the potential to correct those errors in real-time. For instance, eliminating small bubbles, that would otherwise be unseen in luminometers, increases the accuracy and precision of luminescent measurements. It should also be noted that this type of chemiluminescent detection is not limited to the ChemiGenius Bioimaging system, but likely many bioimagers that were designed to detect chemiluminescence from Western blots or PCR gels. Individual machine settings will vary, but the novel detection of kinase activity via the Kinase-Glo platform should be widely applicable.

## Appendix A Procedural details for the purification of GSK3 from the skeletal muscle of 13-lined ground squirrels

Frozen skeletal muscle tissue from 13-lined ground squirrels was homogenized 1:5 (v:v) in homogenization buffer (25 mM Tris–HCl, pH 8.0, 2.5 mM EDTA, 2.5 mM EGTA, 15 mM β-mercaptoethanol, 25 mM NaF, and 10% glycerol). The resulting homogenate was then spun at 13, 500 × *g* for 30 min at 5 °C. The supernatant was then isolated and applied to a DEAE Sephadex column (6 cm × 1 cm, h × d) pre-equilibrated in homogenization buffer. This column was subsequently washed with 20 mL of homogenization buffer to remove any unbound proteins, after which a 0–2 M KCl gradient was used to elute bound proteins.

The location of GSK3 within the DEAE Sephadex fractions was determined by Western blot. SDS resolving gels (10% (v/v) acrylamide, 400 mM Tris, pH 8.8, 0.1% (w/v) SDS, 0.2% (w/v) ammonium persulfate (APS), 0.04% (v/v) tetramethylethylenediamine (TEMED)) were prepared with a 5% stacking gel (5% acrylamide, 190 mM Tris, pH 6.8, 0.1% (w/v) SDS, 0.15% (w/v) APS, 0.1% (v/v) TEMED). DEAE fractions were loaded into these gels and separated electrophoretically in SDS-PAGE running buffer (25 mM Tris-base, 190 mM glycine, and 0.1% (w/v) SDS) at 180 V for 45 min. 3 μL of Spectra™ Multicolor Broad Range Protein Ladder was added to one lane of every gel to act as molecular weight markers. Following electrophoresis, proteins were electoblotted onto polyvinylidiene difluoride (PVDF) membranes (Millipore) by wet transfer using transfer buffer (25 mM Tris, pH 8.5, 192 mM glycine, and 20% (v/v) methanol). The electroblotting was run at room temperature for 1.5 h at 160 mA.

Following protein transfer, the PVDF membranes were incubated overnight at 4 °C with GSK3-α/β antibody (Santa Cruz Biotechnology) diluted 1:1000 in Tris-buffered saline with Tween-20 (TBST; 20 mM Tris-base, 140 mM NaCl, 0.05% Tween-20) with a small amount of sodium azide added. After the overnight incubation, membranes were washed with TBST three times for 5 min each, which was followed by incubation with the anti-mouse secondary antibody conjugated with horseradish peroxidase (Bioshop Canada) at a dilution of 1:4000 in TBST. Membranes were incubated at room temperature for 1 h. Membranes were then washed three times for 5 min each time, and signal was then detected using enzymatic chemiluminescence (ECL). To initiate the membrane exposure 600 μL of hydrogen peroxide was mixed with 600 μL of luminol reagent on the membranes surface for several seconds. The mixture was then poured off and the chemiluminescence was detected ChemiGenius Bioimaging System (Syngene, MD, USA). Fractions identified to have GSK3 within them were then taken, pooled and held on ice until an affinity column could be constructed.

A blue-agarose column (3 cm × 1 cm; h × d) was equilibrated in homogenization buffer prior to the application of the pooled DEAE fractions containing GSK3. This column was then washed with 30 mL of homogenization buffer to wash away unbound proteins, and then proteins were eluted with a 0–2 M KCl gradient. Western blots were again used to identify the fractions that contained GSK3.
